# Clinical Outcome between Ticagrelor versus Clopidogrel in Patients with Acute Coronary Syndrome and Diabetes

**DOI:** 10.1155/2021/5546260

**Published:** 2021-10-15

**Authors:** Peixun He, Xiaolin Luo, Jiabei Li, Yi Li, Xiaozeng Wang, Lan Huang, Jun Jin, Yaling Han

**Affiliations:** ^1^Department of Cardiology, The Second Affiliated Hospital, Army Medical University, Chongqing, China; ^2^Department of Cardiology, General Hospital of Northern Theater Command, Shenyang, China

## Abstract

**Background:**

The increased thrombotic risk in patients with acute coronary syndrome (ACS) and diabetes highlights the need for adequate antithrombotic protection. We aimed to compare the 6-month clinical outcomes between ticagrelor and clopidogrel in patients with ACS and diabetes.

**Methods and Results:**

The study was a single-center, prospective, randomized, open-label, blinded endpoint, and controlled registry trial. A total of 270 ACS patients with diabetes were randomly assigned in a 1 : 1 ratio to either the ticagrelor group or the clopidogrel group. Follow-up was performed for 6 months, and the data on efficacy outcomes and bleeding events were collected. At 6 months, complete follow-up data were available for 266 (98.5%) of 270 patients, and 4 were lost to follow-up. There was no significant difference in the survival rate of the effective endpoints between the ticagrelor group (*n* = 133) and the clopidogrel group (*n* = 133) (HR 0.83, 95% CI 0.44–1.56, *p* = 0.561), but the incidence of bleeding events in the ticagrelor group was higher than that in the clopidogrel group (HR 1.76, 95% CI 1.00–3.10, *p* = 0.049).

**Conclusion:**

Ticagrelor did not improve the composite of nonfatal MI, target vessel revascularization, rehospitalization, stroke, and death from any cause; however, it significantly increased the incidence of bleeding events defined by the Bleeding Academic Research Consortium (BARC) criteria in Chinese patients with ACS and diabetes during the 6-month follow-up compared with clopidogrel.

## 1. Introduction

Acute coronary syndrome (ACS) is one of the major lethal and disabling diseases that affect millions of people worldwide [1]. Following atherosclerotic plaque rupture inside a coronary artery, the initiation of thrombus formation by platelet activation is a major element [2]; ergo, antiplatelet therapy is a landmark treatment strategy for ACS. In China, up to 37% of patients presenting with ACS suffer from diabetes [3]. Among ACS patients, diabetic status was associated with more elements of the ischemic cardiovascular profile [4]; this may be partly related to abnormal platelet function leading to platelet hyperreactivity. Previous studies in patients with ACS and diabetes showed a 1.8-fold increase in cardiovascular deaths and a 1.4-fold increase in myocardial infarctions (MIs) at 2 years compared to nondiabetic patients [5]. Multiple factors, such as hyperglycemia, endothelial dysfunction, and oxidative stress, play a vital role in platelet hyperreactivity in diabetic patients. As such, the higher thrombotic risk in patients with ACS and diabetes highlights the need for adequate antithrombotic protection [6].

Inhibition of platelet aggregation with dual antiplatelet therapy (DAPT) consisting of low-dose aspirin and a P2Y_12_ receptor inhibitor is recognized as a standard treatment for patients after ACS. An impaired response to clopidogrel that occurs in 5% to 44% of patients with diabetes has been reported in multiple pharmacodynamic studies [7]. Prasugrel and ticagrelor, third-generation P2Y_12_ inhibitors, circumvent the clinical limitations of clopidogrel, such as liver metabolism, drug interactions, and polymorphisms in genes encoding platelet receptors, thereby exerting faster and stronger antiplatelet aggregation properties, which suggests their usefulness in patients with ACS and diabetes [8, 9]. Existing guidelines recommend that ACS patients use ticagrelor or prasugrel instead of clopidogrel if there is no contraindication [10, 11]; however, real-world registration data showed that clopidogrel is still widely used [12, 13], which may be, in part, attributable to the higher bleeding risk associated with more potent antithrombosis.

Ticagrelor has been demonstrated to reduce the composite of ischemic events without increasing the overall risk of major bleeding compared with clopidogrel in ACS patients [9]. However, most of the data came from randomized controlled studies in Western countries, and the effectiveness and safety of ticagrelor in East Asian populations have not yet been fully established. The “East Asian Paradox” means that East Asian patients have a lower risk of ischemic events but a higher risk of bleeding complications than non-East Asian patients, despite lower responsiveness to antiplatelet therapy [14, 15], suggesting that Asian patients may not have a better benefit-risk ratio after using more potent P2Y_12_ inhibitors (such as ticagrelor). Therefore, we aimed to compare the 6-month clinical outcomes between ticagrelor and clopidogrel in patients with ACS and diabetes and hopefully provide valuable data in an Asian population.

## 2. Methods

### 2.1. Study Design and Population

The study was a single-center, prospective, randomized, open-label, blinded endpoint, and controlled registry trial carried out at the Institute of Cardiovascular Medicine, the Second Affiliated Hospital, Army Medical University in China. We consecutively enrolled 270 patients with ACS and diabetes. For the study patients, ACS was determined based on the diagnosis of unstable angina or acute MI. Unstable angina pectoris was defined as a patient with symptoms of myocardial ischemia but no increase in troponin, with or without ischemic changes in the electrocardiogram, such as ST-segment depression or new T wave inversion. Acute MI was defined as ST elevation MI or non-ST elevation MI. Each patient underwent percutaneous coronary intervention in the study. Type 2 diabetes was defined as individuals with fasting blood glucose ≥ 126 mg/dL (7.0 mmol/L) or random blood glucose ≥ 200 mg/dL (11.1 mmol/L) or patients with a known history of diabetes who were undergoing hypoglycemic therapy. Fasting was defined as no calorie intake for ≥8 hours. The main exclusion criteria were any contraindications to antiplatelet drugs, the need for oral anticoagulation therapy, the simultaneous use of potent inhibitors or inducers of cytochrome P450 3A, and the combination of chronic infections, malignant tumors, and autoimmune diseases.

This randomized controlled trial is aimed at evaluating the effectiveness and safety of two different antiplatelet strategies. The protocol (Supplemental Figure 1) was approved by the Institutional Ethics Committee, and the trial was registered at http://www.chictr.org.cn (ChiCTR1800015104). The study was conducted in accordance with the Declaration of Helsinki following the Good Clinical Practice Guidelines. Fifty-seven patients refused to enter after reading the informed consent form, and ultimately, informed consent forms from 270 eligible participants were obtained. This report complied with the Consolidated Standards of Reporting Trial (CONSORT) statement.

### 2.2. Randomization and Treatment Groups

Eligible patients were randomly assigned to the ticagrelor group or the clopidogrel group at a 1 : 1 ratio through an interactive voice response or network response system. Randomization codes were generated in blocks of constant size. Randomization was carried out, and once a patient was included, administration of the study regimen started. The treatment groups were allocated in an open-label manner. Patients in the ticagrelor group received a loading dose of 180 mg, followed by oral ticagrelor at 90 mg, taken twice per day, while patients in the clopidogrel group who had not received a loading dose and had not taken clopidogrel for at least 5 days before randomization received a loading dose of 300 mg, followed by a dosage of 75 mg per day, or a maintenance dosage of 75 mg per day. During the entire study period, all patients received oral aspirin at 100 mg once per day.

### 2.3. Data Collection

Data including the patients' baseline characteristics, past medical history, risk factors, clinical diagnosis, medications at the time of admission and discharge, in-hospital biochemistry, and interventions/procedures were collected from questionnaires by a specially trained staff worker. Percutaneous coronary intervention (PCI) was performed in a conventional manner. All patients were given antiplatelet drugs before the intervention, with aspirin and clopidogrel or ticagrelor, according to the principle of randomization.

### 2.4. Follow-Up and Clinical Outcomes

Follow-up was performed for 6 months by phone interview or personal contact, and data on efficacy (nonfatal MI, target vessel revascularization, rehospitalization, stroke, and death from any cause) and safety (bleeding events) outcomes were collected. MI was defined according to the fourth universal definition proposed in 2018. Target vessel revascularization was defined as percutaneous revascularization or bypass surgery for the target lesion or any arterial segment containing the target lesion. Rehospitalization was defined as hospitalization for unstable angina. Ischemic stroke was characterized by the onset of neurological dysfunction caused by focal brain, spinal cord, or retinal infarction. The Bleeding Academic Research Alliance (BARC) standards were used to evaluate bleeding events.

### 2.5. Statistical Analysis

Continuous variables are represented by the median (interquartile range, IQR), and categorical variables are represented by *n* (%). As appropriate, the Mann-Whitney *U* test, Pearson's *χ*^2^ test, the continuity correction test, or Fisher's exact test was used to compare the differences between the ticagrelor group and the clopidogrel group. Binary logistic regression models were employed to identify the independent risk factors. Cox proportional hazards regression analysis was used to explore the correlation between the different antiplatelet regimens and clinical outcomes. Variables considered to be clinically relevant or that showed statistically univariate significance with clinical outcomes (*p* < 0.20) were included in the multivariate regression model. To ensure the simplicity of the final model, the variables were carefully selected based on the number of available events, and co-linearity was avoided. The odds ratio (OR) or hazard ratio (HR) was used to clarify the relations between risk factors and clinical outcomes. *p* < 0.05 was considered statistically significant. Statistical analyses were performed using SPSS software version 20 (IBM Corp., Armonk, NY, USA). Statistical power calculations were performed using PASS software, version 11 (NCSS, LLC, Kaysville, UT, USA).

## 3. Results

### 3.1. Patients

A total of 270 ACS patients with diabetes were enrolled in the current study between October 2017 and March 2019. The 6-month follow-up period ended in September 2019. The recruited patients were randomly divided into the clopidogrel group (*n* = 135) and the ticagrelor group (*n* = 135). At 6 months, 266 (98.5%) of the 270 patients had complete follow-up data available, and 4 patients (2 in the ticagrelor group and 2 in the clopidogrel group) were lost to follow-up due to missing phone numbers or their own reasons (Supplemental Figure 1). Both the clopidogrel group (*n* = 133) and the ticagrelor group (*n* = 133) were well balanced in almost all baseline characteristics (Table 1), including demographics, medical history, medication, biomedical indicators, and the results of coronary angiography, although patients with hypertension were more likely to be in the clopidogrel group (*p* = 0.038). There seemed to be more patients in the ticagrelor group with chronic kidney disease, but the difference was not significant (*p* = 0.053).

### 3.2. Clinical Outcomes

At 6 months, the proportion of successful revascularizations in the ticagrelor group was lower than that in the clopidogrel group in terms of efficacy outcomes, but there was no significant difference between the two groups (14.3% vs. 16.5%, *p* = 0.610). For the safety outcome, the total number of bleeding events defined by BARC in the ticagrelor group was slightly more than that in the clopidogrel group, although there was no significant difference (24.1% vs. 15.8%, *p* = 0.091); particularly in the BARC type 2 group, the bleeding risk in the ticagrelor group showed a tendency to increase (6.0% vs. 1.5%, *p* = 0.053) (Table 2).

### 3.3. Risk Factors of Outcomes

The demographic characteristics, medical history, medication, biomedical indicators, the results of coronary angiography, and grouping were included in the univariate logistic regression model analysis, and age, hypertension, liver insufficiency, hemoglobin, and estimated glomerular filtration rate (eGFR) were potential influencing factors for the composite effectiveness endpoint (Supplemental Table 1). Then, through the multivariate model for calibration analysis, we found that liver insufficiency was an independent risk factor that affected the effectiveness outcomes (*p* = 0.006) (Table 3). The same logistic regression model was used to analyze the possible risk factors for the bleeding endpoints (Table 4 and Supplemental Table 2).

### 3.4. Survival Analysis

Univariate and multivariate Cox proportional hazards regression models were used serially to identify the factors affecting the survival outcome of the efficacy and safety endpoints, and the included variables were the same as those mentioned above. Age, hypertension, liver insufficiency, hemoglobin, and eGFR were possible confounding factors for the survival outcome of the effectiveness endpoints (Supplemental Table 1), and liver insufficiency (*p* = 0.002) and eGFR (*p* = 0.026) were found to be independent factors influencing the survival of the effectiveness endpoints (Table 3) in the multivariate model. Through the same statistical model, the treatment grouping was demonstrated to be an independent factor that affected the survival outcome of the bleeding endpoints defined by BARC (Table 4 and Supplemental Table 2).

**Figure 1:**
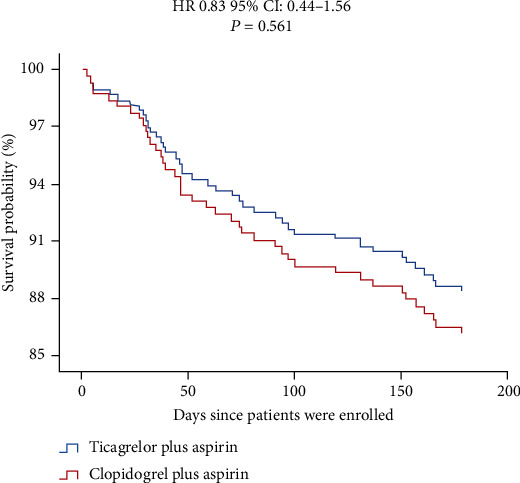
Event-free survival for the composite of efficacy outcomes in ACS patients with diabetes. There was no significant difference in the survival outcomes of MACEs between the ticagrelor group (blue line) and the clopidogrel group (red line) (HR 0.83, 95% CI 0.44–1.56, *p* = 0.561).

**Figure 2:**
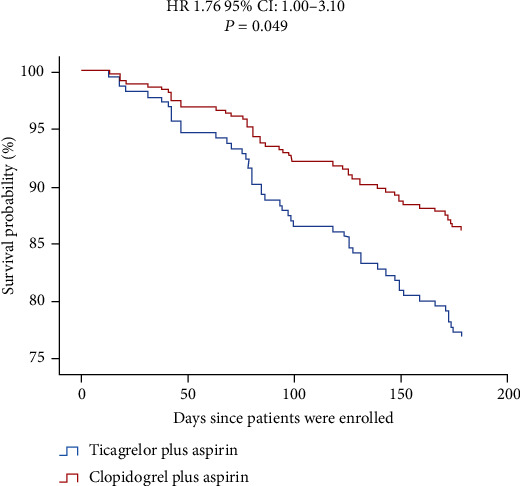
Event-free survival for bleeding events defined by the BARC criteria in ACS patients with diabetes. The incidence of bleeding events in the ticagrelor group (red line) was higher than that in the clopidogrel group (blue line) (HR 1.76, 95% CI 1.00–3.10, *p* = 0.049).

Based on the Cox survival regression analysis model, we further compared the differences in the 6-month follow-up endpoint events between the two treatment groups. The results showed that there was no significant difference in the survival rate of the effectiveness endpoint between the two groups (HR 0.83, 95% CI 0.44–1.56, *p* = 0.561) (Figure 1), but the incidence of bleeding events in the ticagrelor group was higher than that in the clopidogrel group (HR 1.76, 95% CI 1.00–3.10, *p* = 0.049) (Figure 2).

## 4. Discussion

The study was conducted to compare the 6-month clinical outcomes between the clopidogrel and ticagrelor groups in Asian patients with ACS and diabetes. The main findings of our study on a Chinese population were that ticagrelor did not improve the survival rate of efficacy outcomes (composite of nonfatal MI, target vessel revascularization, rehospitalization, stroke, and death from any cause) but increased the prevalence of bleeding events defined by BARC criteria in patients with ACS and diabetes compared to clopidogrel.

Diabetes has a clear negative impact on the clinical outcome of ACS patients [16]. Although the underlying causes may be multifaceted [17, 18], platelet insufficiency is common in diabetic patients, in whom hyperglycemia, endothelial and vascular damage, and chronic proinflammatory and prothrombotic environments promote platelet activation [19, 20]. Highly reactive platelets are a key factor that accelerates atherosclerosis and leads to adverse ischemic or thrombotic events [6, 21]. Therefore, the strength of the antiplatelet regimen is very important for patients with ACS and diabetes [22]. The “East Asian Paradox” refers to the low potential risk of ischemic events, but the high risk of bleeding in East Asian populations, which poses a challenge to the current “one size fits all” antiplatelet therapy strategy for ACS patients [23–25]. In dealing with the specific population of patients with ACS combined with diabetes, it is necessary to pay attention to the more complex balance between ischemia and bleeding complications and further optimize the antiplatelet strategy, which is conducive to improving patient outcomes.

At present, the results of studies on optimized dual antithrombotic regimens for patients with ACS and diabetes are controversial. The PLATO study shows that compared with clopidogrel, ticagrelor treatment significantly reduced the risk of major adverse cardiovascular events (MACEs) in patients with ACS and played an effective role in antithrombosis without significantly increasing the risk of major bleeding [26]. A substudy of PLATO showed that ticagrelor showed a better benefit-risk value than clopidogrel regardless of diabetes status and blood sugar control [9]. In the subgroup analysis of the TRITON-TIMI 38 trial, prasugrel, another effective ADP P2Y12 antagonist, reduced the risk of cardiovascular death, myocardial infarction, or stroke by 4.8% compared with clopidogrel (30% relative) [8].

However, some studies have different conclusions. Spoendlin et al. conducted a cohort study using United States commercial claims data (2009–2015) in ACS patients with diabetes and revealed that the prasugrel group had better cardiovascular outcomes despite a higher risk of short-term bleeding. No significant difference was found in two different comparisons between the ticagrelor and clopidogrel groups or between the prasugrel and ticagrelor groups [27]. Data from the Korean Acute MI Registry-National Institutes of Health showed that in MI patients with diabetes undergoing PCI, the use of prasugrel/ticagrelor (*n* = 1000) did not improve the composite of cardiac death, recurrent MI, or stroke but significantly increased the number of major bleeding events compared with clopidogrel treatment (*n* = 2985) [28]. Additionally, our research has obtained a similar result in that ticagrelor increased the incidence of bleeding events without improving the efficacy outcomes, suggesting that East Asian patients may potentially be different from Western patients. Furthermore, Goto et al. performed a study on the optimized antiplatelet regimen of ACS patients with diabetes in Japan, Taiwan, and South Korea and found that the number of major bleeding events in the ticagrelor treatment group was higher, albeit not significantly. However, there was no significant difference in ischemia risk between the ticagrelor and clopidogrel groups [29]. Park et al. compared the treatment differences between ticagrelor and clopidogrel in Korean acute myocardial infarction (AMI) patients, 22% of whom had diabetes, and found that ticagrelor did not reduce the risk of ischemia but increased the 6-month Thrombolysis in Myocardial Infarction (TIMI) major bleeding events [30].

Furthermore, in the current study, age, hypertension, liver insufficiency, hemoglobin, and eGFR were found to be potential influencing factors for the composite effectiveness endpoint in patients with ACS and diabetes in univariate logistic regression model analysis, and liver insufficiency was an independent risk factor that affected the effectiveness outcomes in the multivariate model for calibration analysis. In addition, there are many factors that may affect the prognosis of patients who underwent PCI, including their own conditions, the types and specifications of implanted devices, and the choices and timing of medications. A meta-analysis of 64 randomized controlled trials and 102 735 patients after approximately 20 months of follow-up showed that the type of stent implanted seems to have a partial impact on the risk of adverse events in patients and that different treatment durations of DAPT are also related to bleeding risk [31].

Atherothrombosis is a highly complex process [32], and a considerable amount of data shows that ethnic differences have an impact on thrombosis, which is reflected by coagulation, fibrinolysis, and inflammation markers [33]. East Asian patients have a low body mass index (BMI) and are significantly different from Western patients in terms of thrombosis, platelet receptor inhibition, and susceptibility to bleeding risk. Therefore, East Asian patients have a higher risk of bleeding associated with antiplatelet therapy during antithrombotic therapy [23–25]. This may explain why our results did not reveal a difference in effectiveness between the two groups but showed an increased risk of bleeding in the ticagrelor group compared to the clopidogrel group. Most of the current trials evaluating the clinical efficacy and safety of P2Y12 receptor potent inhibitors (ticagrelor/prasugrel) in ACS patients with diabetes do not include a sufficient number of East Asian participants, and it is difficult to draw trustworthy conclusions [15]. Therefore, before using the powerful P2Y12 inhibitors recommended by studies conducted on Western populations to treat patients with ACS complicated with diabetes, more specific studies on East Asian populations in this field are required.

This study has several limitations. First, although our study is based on prospective, randomized, open-label, blinded endpoints, and controlled registries, it is a small-scale, single-center study, and the small sample size may limit the power to detect differences in clinical outcomes. Second, we did not include information on the lifestyle of the patients regarding the type of diet and frequency of exercise per week or the frequencies of drinking and smoking. This lack of information seems slightly rudimentary in terms of lifestyle surveys. Third, middle-aged and elderly heart disease patients usually have other diseases, such as diabetes, hypertension, and gout, which causes them to take multiple drug treatments. Actually, the impact of polypharmacy with the varied disease backgrounds and other complications the patients have made it difficult to arrive at a definitive conclusion of the study. Fourth, the duration of follow-up was limited, and it is possible that a longer follow-up period could have displayed significantly different outcomes between the ticagrelor and clopidogrel groups of ACS patients with diabetes.

## 5. Conclusion

Our study shows that ticagrelor did not improve the composite of nonfatal MI, target vessel revascularization, rehospitalization, stroke, and death from any cause; however, ticagrelor significantly increased the number of bleeding events defined by the BARC criteria in Chinese patients with ACS and diabetes during the 6-month follow-up compared with clopidogrel. These results seem to suggest the need to transform antiplatelet strategies for the treatment of ACS patients with diabetes from “one guideline suitable for all races” to “racially differentiated antiplatelet therapy,” but more dedicated studies in East Asian populations are required.

## Figures and Tables

**Table 1:** Baseline characteristics of ACS patients with diabetes.

	Total (*n* = 266)	Ticagrelor plus aspirin (*n* = 133)	Clopidogrel plus aspirin (*n* = 133)	*p* value
Age, years	64.0 (57.0–69.0)	64.0 (56.0–68.0)	64.0 (57.0–69.0)	0.671
Males, *n* (%)	86 (32.3%)	39 (29.3%)	47 (35.3%)	0.294
BMI, kg/m^2^	24.8 (22.9–27.3)	24.8 (23.0–27.6)	24.8 (22.8–26.8)	0.404
Current smoker, *n* (%)	141 (53.0%)	73 (54.9%)	68 (51.1%)	0.539
Current drinking, *n* (%)	107 (40.2%)	52 (39.1%)	55 (41.4%)	0.708
UAP, *n* (%)	199 (74.8%)	96 (72.2%)	103 (77.4%)	0.323
STEMI, *n* (%)	32 (12.0%)	19 (14.3%)	13 (9.8%)	0.258
NSTEMI, *n* (%)	35 (13.2%)	18 (13.5%)	17 (12.8%)	0.856
Heart rate, bpm	78.0 (70.0–87.0)	78.0 (71.0–88.0)	78.0 (68.5–86.0)	0.402
SBP, mmHg	131.5 (117.0–144.3)	132.0 (115.0–149.5)	131.0 (118.0–142.0)	0.682
DBP, mmHg	73.0 (63.0–82.0)	73.0 (61.5–82.0)	73.0 (65.0–81.5)	0.687
History				
Previous MI, *n* (%)	34 (12.8%)	21 (15.8%)	12 (9.8%)	0.142
Previous coronary stent implantation, *n* (%)	46 (17.3%)	26 (19.5%)	20 (15.0%)	0.331
Previous GI bleeding, *n* (%)	8 (3.0%)	4 (3.0%)	4 (3.0%)	1.000
Hypertension, *n* (%)	176 (66.2%)	80 (60.2%)	96 (72.2%)	0.038
Hyperuricemia, *n* (%)	15 (5.6%)	6 (4.5%)	9 (6.8%)	0.425
Hyperlipemia, *n* (%)	57 (21.4%)	28 (21.1%)	29 (21.8%)	0.881
Liver insufficiency, *n* (%)	11 (4.1%)	8 (63.0%)	3 (2.3%)	0.124
Chronic kidney disease, *n* (%)	30 (11.3%)	20 (15.0%)	10 (7.5%)	0.053
Ischemic stroke, *n* (%)	22 (8.3%)	9 (6.8%)	13 (9.8%)	0.373
Medication				
Statins, *n* (%)	262 (98.5%)	132 (99.2%)	130 (97.7%)	0.314
Nitrate, *n* (%)	66 (24.8%)	32 (24.1%)	34 (25.6%)	0.776
Beta blockers, *n* (%)	198 (74.7%)	97 (72.9%)	101 (76.5%)	0.502
RAAS inhibitors, *n* (%)	192 (72.5%)	91 (68.9%)	101 (75.9%)	0.202
Calcium channel blockers, *n* (%)	71 (26.7%)	33 (24.8%)	38 (28.6%)	0.488
Proton pump inhibitors, *n* (%)	148 (55.6%)	76 (57.2%)	72 (54.1%)	0.622
Insulin, *n* (%)	95 (35.7%)	52 (39.1%)	43 (32.3%)	0.249
Metformin, *n* (%)	120 (45.1%)	57 (42.9%)	63 (47.4%)	0.460
Acarbose, *n* (%)	74 (27.8%)	33 (24.8%)	41 (30.8%)	0.274
Other hypoglycemic agents, *n* (%)	97 (36.5%)	42 (31.6%)	55 (41.4%)	0.098
Biomedical indicators				
Leukocyte, 10^9^/L	6.9 (5.9–8.2)	7.0 (5.9–8.3)	6.8 (5.9–8.1)	0.534
Hemoglobin, g/L	130.0 (118.0–142.0)	131.0 (116.5–143.0)	129.0 (119.0–141.5)	0.802
Platelets, 10^9^/L	189.5 (150.0–220.3)	189.0 (155.0–224.0)	188.0 (146.5–219.0)	0.597
Mean platelet volume, fL	11.5 (10.4–12.6)	11.4 (10.4–12.5)	11.6 (10.4–12.6)	0.413
Platelet distribution width, fL	16.0 (13.8–17.1)	15.5 (13.7–17.0)	16.2 (14.0–17.3)	0.124
ALT, U/L	23.7 (17.1–36.3)	23.7 (17.4–37.5)	23.7 (16.8–36.3)	0.773
AST, U/L	21.1 (16.1–28.3)	21.1 (16.3–29.2)	20.6 (15.6–26.1)	0.369
Creatinine, *μ*mol/L	76.2 (63.0–88.4)	77.3 (65.0–88.7)	74.5 (61.3–88.4)	0.317
eGFR, mL/min	88.0 (73.0–96.0)	88.0 (74.5–95.0)	88.0 (73.0–97.0)	0.906
Total cholesterol, mmol/L	3.7 (3.1–4.5)	3.7 (3.1–4.5)	3.7 (3.1–4.5)	0.437
Triglyceride, mmol/L	1.5 (1.1–2.1)	1.4 (1.0–1.9)	1.5 (1.2–2.1)	0.085
Glycosylated hemoglobin, %	7.7 (6.8–8.9)	7.7 (6.8–9.2)	7.7 (6.8–8.7)	0.565
Coronary angiography				
Single-vessel disease, *n* (%)	42 (15.8%)	18 (13.5%)	24 (18.0%)	0.313
Double-vessel disease, *n* (%)	105 (39.5%)	48 (36.1%)	57 (42.9%)	0.258
Triple-vessel disease, *n* (%)	119 (44.7%)	67 (50.4%)	52 (39.1%)	0.064

Data were expressed as *n* (%) and median (IQR). IQR: interquartile range; *p* value, Mann-Whitney *U* test, or Pearson chi-square test; ACS: acute coronary syndrome; ALT: alanine aminotransferase; AST: aspartate aminotransferase; BMI: body mass index; DBP: diastolic blood pressure; eGFR: estimated glomerular filtration rate; GI: gastrointestinal; MI: myocardial infarction; NSTEMI: non-ST-segment elevation myocardial infarction; STEMI: ST-segment elevation myocardial infarction; SBP: systolic blood pressure; RAAS: renin-angiotensin-aldosterone system; UAP: unstable angina pectoris.

**Table 2:** Clinical outcomes in ACS patients with diabetes.

	Total (*n* = 266)	Ticagrelor plus aspirin (*n* = 133)	Clopidogrel plus aspirin (*n* = 133)	*p* value
Efficacy outcome				
Composite endpoint	41 (15.4%)	19 (14.3%)	22 (16.5%)	0.610
Nonfatal MI	0 (0.0%)	0 (0.0%)	0 (0.0%)	—
Target vessel revascularization	15 (5.6%)	5 (3.8%)	10 (7.5%)	0.184
Rehospitalization	17 (6.4%)	9 (6.8%)	8 (6.0%)	0.802
Ischemic stroke	3 (1.1%)	1 (0.8%)	2 (1.5%)	1.000
Death from any cause	6 (2.3%)	4 (3.0%)	2 (1.5%)	0.680
BARC type				
BARC 1~5	53 (19.9%)	32 (24.1%)	21 (15.8%)	0.091
BARC 1	38 (14.3%)	21 (15.8%)	17 (12.8%)	0.483
BARC 2	10 (3.8%)	8 (6.0%)	2 (1.5%)	0.053
BARC 3	4 (1.5%)	2 (1.5%)	2 (1.5%)	1.000
BARC 4	0 (3.5%)	0 (0.0%)	0 (0.0%)	—
BARC 5	1 (0.4%)	1 (0.8%)	0 (0.0%)	1.000

Data were expressed as *n* (%) and median (IQR). IQR: interquartile range; *p* value, Pearson chi-square test, continuity correction test, or Fisher's exact test; composite endpoints included MI, revascularization, rehospitalization for angina, stroke, and death from any cause; BARC: Bleeding Academic Research Consortium definition for bleeding; MI: myocardial infarction.

**Table 3:** Risk factors for the composite efficacy outcomes of ACS patients with diabetes in multivariable analysis.

Variable	Multivariable OR (95% CI)	*p*1 value	Multivariable OR (95% CI)	*p*2 value
Age, years	1.04 (0.98–1.09)	0.186	1.03 (0.98–1.08)	0.267
History				
Hypertension	2.14 (0.90–5.09)	0.085	1.85 (0.84–4.05)	0.125
Liver insufficiency	6.55 (1.73–24.78)	0.006	4.52 (1.74–11.77)	0.002
Biomedical indicator				
Hemoglobin	0.99 (0.98–1.01)	0.184	0.99 (0.98–1.00)	0.181
eGFR	0.98 (0.97–1.00)	0.069	0.98 (0.97–1.00)	0.026
Grouping (ticagrelor vs. clopidogrel)	—	—	0.83 (0.44–1.56)	0.561

95% CI: 95% confidence interval; OR: odds ratio; *p*1: logistic regression analysis; *p*2: Cox survival analysis; BMI: body mass index; MI: myocardial infarction; GI: gastrointestinal; RAAS: renin-angiotensin-aldosterone system; ALT: alanine aminotransferase; eGFR: estimated glomerular filtration rate.

**Table 4:** Risk factors for bleeding events defined by the BARC criteria in ACS patients with diabetes in multivariable analysis.

Variable	Multivariable OR (95% CI)	*p* value	Multivariable OR (95% CI)	*p* value
Age, years	0.97 (0.93–1.00)	0.056	0.97 (0.94–1.00)	0.068
History				
Chronic kidney disease	0.37 (0.11–1.29)	0.120	0.39 (0.12–1.26)	0.117
Biomedical indicator				
Triglyceride	1.13 (0.94–1.35)	0.204	1.11 (0.98–1.27)	0.107
Grouping (ticagrelor vs. clopidogrel)	1.80 (0.95–3.41)	0.070	1.76 (1.00–3.10)	0.049

95% CI: 95% confidence interval; OR: odds ratio; *p*1: logistic regression analysis; *p*2: Cox survival analysis; BMI: body mass index; MI: myocardial infarction; GI: gastrointestinal; RAAS: renin-angiotensin-aldosterone system; ALT: alanine aminotransferase; eGFR: estimated glomerular filtration rate.

## Data Availability

The data that support the findings of this study are available from the corresponding author upon reasonable request.

## Supplementary Materials

Supplementary Materials Supplemental Figure 1: flow chart of the study. Supplemental Table 1: risk factors for the composite efficacy outcomes of ACS patients with diabetes in univariable analysis. Supplemental Table 2: risk factors for the bleeding events defined with BARC criteria of ACS patients with diabetes in univariable analysis.
