# Supplementary material for: Clinical Outcome between Ticagrelor versus Clopidogrel in Patients with Acute Coronary Syndrome and Diabetes
Source: Cardiovasc Ther. 2021 Oct 15;2021:5546260. doi: 10.1155/2021/5546260 (PMC8536459; doi:10.1155/2021/5546260)
Supplement: Supplementary Materials — Supplemental Figure 1: flow chart of the study. Supplemental Table 1: risk factors for the composite efficacy outcomes of ACS patients with diabetes in univariable analysis. Supplemental Table 2: risk factors for the bleeding events defined with BARC criteria of ACS patients with diabetes in univariable analysis. [file 5546260.f1.pdf]

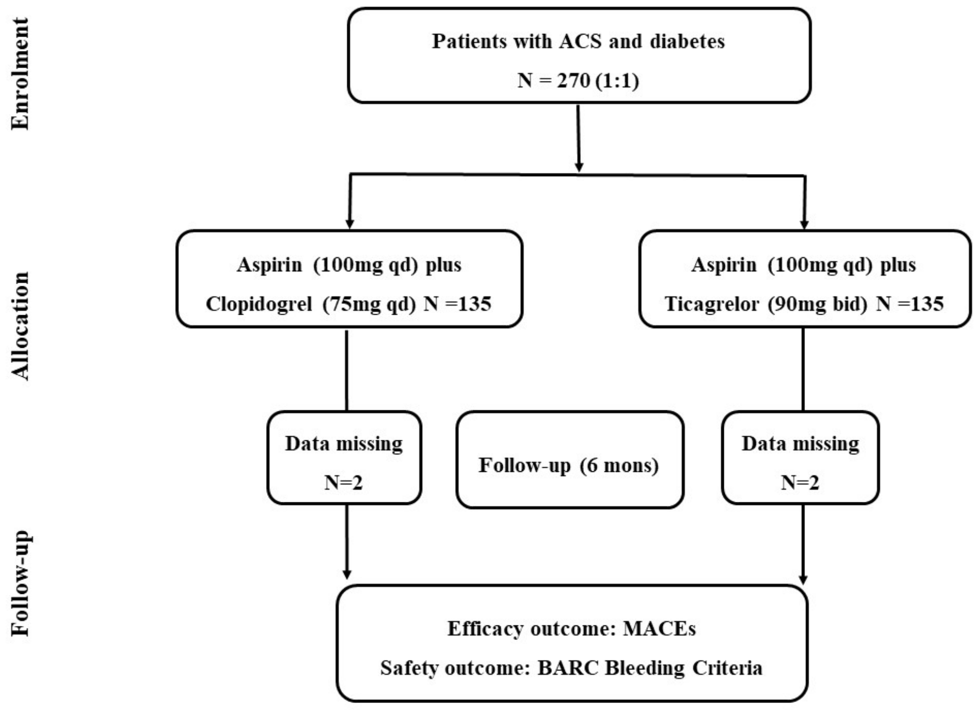

SUPPLEMENTAL FIGURE 1: Flow chart of the study.

SUPPLEMENTAL TABLE 1: Risk factors for the composite efficacy outcomes of ACS patients with diabetes in univariable analysis.

Variable	Univariable OR (95% CI)	P1 value	Univariable OR (95% CI)	P2 value
Age, years	1.06 (1.01-1.11)	0.012	1.06 (1.01-1.11)	0.013
Gender (male vs female)	0.80 (0.40-1.60)	0.527	0.83 (0.44-1.56)	0.558
BMI, kg/m^2	0.94 (0.85-1.05)	0.290	0.95 (0.86-1.05)	0.315
Current smoking	0.82 (0.42-1.59)	0.556	0.85 (0.46-1.56)	0.594
Current drinking	0.83 (0.42-1.66)	0.606	0.86 (0.46-1.63)	0.643
History				
Hypertension	2.37 (1.04-5.36)	0.039	2.20 (1.02-4.77)	0.045
Ischemic stroke	2.24 (0.82-6.11)	0.116	2.00 (0.84-4.74)	0.119
Hyperlipemia	0.35 (0.12-1.03)	0.057	0.38 (0.13-1.06)	0.063
Previous MI	1.86 (0.77-4.45)	0.166	1.87 (0.86-4.04)	0.114
Liver insufficiency	5.07 (1.47-17.48)	0.010	3.81 (1.50-7.72)	0.005
Chronic kidney disease	1.44 (0.55-3.76)	0.462	1.46 (0.61-3.46)	0.395
Previous GI bleeding	0.78 (0.09-6.50)	0.817	0.81 (0.11-5.86)	0.830
Hyperuricemia	2.10 (0.64-6.96)	0.223	2.01 (0.72-5.65)	0.184
Previous coronary stent implantation	1.19 (0.51-2.78)	0.686	1.24 (0.57-2.69)	0.581
Medication				
Proton pump inhibitors	1.30 (0.66-2.56)	0.455	1.23 (0.66-2.31)	0.514
Insulin	1.34 (0.68-2.63)	0.405	1.30 (0.70-2.41)	0.413

Metformin	0.66 (0.33-1.31)	0.235	0.67 (0.35-1.26)	0.208
Beta blockers	0.60 (0.29-1.22)	0.159	0.65 (0.34-1.24)	0.194
Statins	0.54 (0.06-5.33)	0.598	0.65 (0.09-4.72)	0.670
RAAS inhibitors	1.21 (0.56-2.62)	0.623	1.16 (0.57-2.36)	0.688
Biomedical indicator				
Leucocyte	1.09 (0.95-1.25)	0.201	1.08 (0.96-1.22)	0.194
Hemoglobin	0.99 (0.97-1.00)	0.032	0.99 (0.98-1.00)	0.020
Platelets	1.00 (0.99-1.00)	0.419	1.00 (0.99-1.00)	0.438
Mean platelet volume	0.98 (0.80-1.20)	0.861	0.99 (0.82-1.19)	0.877
Platelet distribution width	0.94 (0.83-1.06)	0.294	0.94 (0.84-1.05)	0.258
ALT	1.01 (1.00-1.02)	0.092	1.01 (1.00-1.02)	0.070
eGFR	0.98 (0.96-0.99)	0.005	0.98 (0.97-0.99)	0.001
Triglyceride	0.87 (0.63-1.20)	0.396	0.88 (0.65-1.19)	0.405
Total cholesterol	0.75 (0.53-1.06)	0.105	0.77 (0.55-1.06)	0.108
Glycosylated hemoglobin	0.92 (0.73-1.16)	0.483	0.91 (0.73-1.14)	0.426
Coronary arteriography				
Single-vessel disease	ref	0.113	ref	0.105
Double-vessel disease	2.00 (0.54-7.35)	0.297	1.95 (0.56-6.79)	0.294
Triple-vessel disease	3.28 (0.94-11.54)	0.064	3.11 (0.94-10.34)	0.064
Grouping (ticagrelor vs clopidogrel)	0.84 (0.43-1.64)	0.611	0.87 (0.47-1.61)	0.656

95% CI, 95% confidence interval; OR, Odds ratio; P1, Logistic regression analysis; P2, COX survival analysis;

BMI, Body mass index; MI, Myocardial infarction; GI, Gastrointestinal; RAAS, Renin-angiotensin-aldosterone system; ALT, Alanine aminotransferase; eGFR, Estimated glomerular filtration rate.

SUPPLEMENTAL TABLE 2: Risk factors for the bleeding events defined with BARC criteria of ACS patients with diabetes in univariable analysis.

Variable	Univariable OR (95% CI)	P1 value	Univariable OR (95% CI)	P2 value
Age, years	0.97 (0.94-1.00)	0.067	0.97 (0.95-1.00)	0.081
Gender (male vs female)	0.91 (0.48-1.73)	0.777	0.93 (0.53-1.63)	0.797
BMI, kg/m^2	1.01 (0.97-1.05)	0.780	1.00 (0.97-1.04)	0.798
Current smoking	1.20 (0.65-2.20)	0.558	1.16 (0.67-2.00)	0.595
Current drinking	0.79 (0.43-1.48)	0.468	0.81 (0.46-1.41)	0.448
History				
Hypertension	1.23 (0.64-2.36)	0.531	1.19 (0.66-2.14)	0.559
Ischemic stroke	0.88 (0.29-2.73)	0.831	0.91 (0.33-2.53)	0.859
Hyperlipemia	1.42 (0.71-2.85)	0.324	1.38 (0.75-2.54)	0.305
Previous MI	0.84 (0.33-2.16)	0.722	0.78 (0.84-1.96)	0.679
Liver insufficiency	1.54 (0.39-6.01)	0.536	1.57 (0.49-5.03)	0.449
Chronic kidney disease	0.41 (0.12-1.42)	0.160	0.44 (0.14-1.40)	0.164
Previous GI bleeding	0.57 (0.07-4.70)	0.598	0.57 (0.08-4.11)	0.575
Hyperuricemia	0.60 (0.13-2.76)	0.515	0.60 (0.15-2.47)	0.497
Previous coronary stent implantation	1.33 (0.63-2.84)	0.458	1.24 (0.64-2.40)	0.531
Medication				
Proton pump inhibitors	1.27 (0.69-2.35)	0.438	1.22 (0.70-2.11)	0.484
Insulin	1.36 (0.74-2.52)	0.326	1.29 (0.75-2.23)	0.360

Metformin	1.01 (0.55-1.85)	0.978	1.01 (0.59-1.74)	0.960
Beta blockers	0.73 (0.38-1.43)	0.359	0.73 (0.41-1.32)	0.302
Statins	0.74 (0.08-7.29)	0.799	0.82 (0.11-5.96)	0.848
RAAS inhibitors	0.82 (0.42-1.59)	0.562	0.83 (0.46-1.49)	0.521
Biomedical indicators				
Leucocyte	0.99 (0.86-1.13)	0.861	0.99 (0.87-1.12)	0.814
Hemoglobin	1.00 (0.98-1.00)	0.746	1.00 (0.99-1.01)	0.730
Platelets	1.00 (1.00-1.01)	0.727	1.00 (1.00-1.00)	0.871
Mean platelet volume	1.07 (0.89-1.28)	0.492	1.07 (0.91-1.25)	0.450
Platelet distribution width	0.94 (0.84-1.05)	0.246	0.95 (0.86-1.04)	0.256
ALT	1.00 (0.98-1.01)	0.631	1.00 (0.99-1.01)	0.675
eGFR	1.01 (0.99-1.03)	0.204	1.01 (1.00-1.03)	0.195
Triglyceride	0.87 (0.63-1.20)	0.160	1.14 (0.99-1.31)	0.063
Total cholesterol	0.96 (0.73-1.26)	0.781	0.97 (0.75-1.26)	0.822
Glycosylated hemoglobin	1.04 (0.85-1.28)	0.680	1.03 (0.86-1.24)	0.727
Coronary arteriography				
Single-vessel disease	ref	0.613	ref	0.655
Double-vessel disease	1.59 (0.60-4.25)	0.355	1.48 (0.60-3.64)	0.399
Triple-vessel disease	1.60 (0.61-4.21)	0.345	1.50 (0.62-3.66)	0.371
Grouping (ticagrelor vs clopidogrel)	1.69 (0.92 -3.12)	0.093	1.70 (0.98 - 2.95)	0.059

95% CI, 95% confidence interval; OR, Odds ratio; P1, Logistic regression analysis; P2, COX survival analysis;

BMI, Body mass index; MI, Myocardial infarction; GI, Gastrointestinal; RAAS, Renin-angiotensin-aldosterone system; ALT, Alanine aminotransferase; eGFR, Estimated glomerular filtration rate.
